# Efficacy of continuous EGFR-inhibition and role of Hedgehog in EGFR acquired resistance in human lung cancer cells with activating mutation of EGFR

**DOI:** 10.18632/oncotarget.15479

**Published:** 2017-02-18

**Authors:** Carminia Maria Della Corte, Umberto Malapelle, Elena Vigliar, Francesco Pepe, Giancarlo Troncone, Vincenza Ciaramella, Teresa Troiani, Erika Martinelli, Valentina Belli, Fortunato Ciardiello, Floriana Morgillo

**Affiliations:** ^1^ Oncologia Medica, Dipartimento Medico-Chirurgico di Internistica Clinica e Sperimentale “F. Magrassi e A. Lanzara,” Università degli studi della Campania “Luigi Vanvitelli”, Naples, Italy; ^2^ Dipartimento di Sanità Pubblica, Università degli Studi di Napoli Federico II, Naples, Italy

**Keywords:** EGFR inhibitors, lung cancer, cell signalling, hedgehog, EMT

## Abstract

**Purpose:**

The aim of this work was to investigate the efficacy of sequential treatment with first-, second- and third-generation epidermal growth factor receptor (EGFR) tyrosine kinase inhibitors and the mechanisms of acquired resistance occurring during the sequential use of these inhibitors.

**Experimental design:**

We developed an *in vivo* model of acquired resistance to EGFR-inhibitors by treating nude mice xenografted with HCC827, a human non-small-cell lung cancer (NSCLC) cell line harboring *EGFR* activating mutation, with a sequence of first-generation EGFR tyrosine kinase inhibitors (EGFR-TKIs) (erlotinib and gefitinib), of second-generation EGFR-TKI (afatinib) plus/minus the anti-EGFR monoclonal antibody cetuximab, and of third-generation EGFR-TKI (osimertinib).

**Results:**

HCC827-derived xenografts and with acquired resistance to EGFR-inhibitors were sensitive to the sequential use of first-, second- and third-generation EGFR-TKIs. Continuous EGFR inhibition of first-generation resistant tumors by sequential treatment with afatinib plus/minus cetuximab, followed by osimertinib, represented an effective therapeutic strategy in this model. Whereas T790M resistance mutation was not detected, a major mechanism of acquired resistance was the activation of components of the Hedgehog (Hh) pathway. This phenomenon was accompanied by epithelial-to-mesenchymal transition. Cell lines established *in vitro* from gefitinib-, or afatinib- or osimertinib-resistant tumors showed metastatic properties and maintained EGFR-TKIs resistance *in vitro*, that was reverted by the combined blockade of Hh, with the selective SMO inhibitor sonidegib, and EGFR.

**Conclusions:**

*EGFR*-mutant NSCLC can benefit from continuous treatment with EGFR-inhibitors, indepenently from mechanisms of resistance. In a complex and heterogenous scenario, Hh showed an important role in mediating resistance to EGFR-inhibitors through the induction of mesenchymal properties.

## INTRODUCTION

The epidermal growth factor receptor (EGFR) is an established target for anti-cancer treatment in non-small cell lung cancer (NSCLC). Tumors containing activating *EGFR* mutations (deletion in exon 19 or an L858R point mutation), which account for about 16% of advanced NSCLC patients, result sensitive to the first- and second-generation EGFR tyrosine kinase inhibitors (EGFR-TKIs) gefitinib, erlotinib, and afatinib, respectively [1, 2].

However, EGFR-TKIs therapies are not curative: most patients with *EGFR* mutant NSCLC treated with EGFR-TKIs develop resistance within 9–14 months [1–3].

Mechanisms of resistance to first-generation EGFR-TKIs are widely known and include for the majority of cases the onset of the second-site *EGFR* mutation substituting threonine for methionine at position 790 in exon 20 (T790M), the activation of other cellular signaling such as MET [4], ERBB2, AXL [5], Hedgehog (Hh) [6] or of downstream escape mediators (BRAF, PIK3CA) and histological changes as epithelial-to-mesenchymal transition (EMT) and small cell lung cancer (SCLC) [7, 8].

A strategy that has demonstrated significant activity in overcoming acquired resistance to erlotinib and gefitinib is the dual inhibition of EGFR with the second-generation EGFR tyrosine kinase inhibitor (EGFR-TKI) afatinib and the anti-EGFR monoclonal antibody cetuximab, which induces tumor regression of T790M+ transgenic mouse lung tumors [9, 10]. The addition of cetuximab to afatinib results in simultaneous depletion of phospho- and total EGFR levels [9]. In a subsequent phase Ib clinical trial of afatinib *plus* cetuximab, a 29% response rate was observed in patients with acquired resistance to gefitinib or erlotinib, regardless of T790M status [10]. Thus, a substantial fraction of *EGFR*-mutant tumors remain dependent on the EGFR signaling axis for survival even after acquisition of resistance to first-generation EGFR-TKIs. Although resistance to afatinib *plus* cetuximab has already been observed in patients, a complete understanding of the spectrum of resistance mechanisms is currently lacking. A recent breakthrough in the treatment of *EGFR* T790M mutant cancers occurred with the development of mutant selective pyrimidine based third-generation EGFR-TKIs, which include the WZ4002, CO-1686, osimertinib and HM61713 inhibitors which have demonstrated tumor responses in > 50% of patients harboring *EGFR* T790M mutation [11–14]. Additionally, their reduced affinity for wild type *EGFR* provokes less toxicity than other EGFR-TKIs. However, resistance will also occur for this class of EGFR inhibitors [11]. As these new compounds become widely available for clinical use, patients will be treated with multiple lines of EGFR-targeted therapies with increasing frequency. However, the effect of sequential treatment with various anti-EGFR agents on tumor evolution and drug resistance in *EGFR*-mutant NSCLC remains to be determined. The aim of the present work was to define the efficacy of sequential treatment with first-, second- and third-generation EGFR-inhibitors and to investigate the potential role of Hh in the acquisition of cancer cell resistance.

## RESULTS

### Therapeutic efficacy of continuing EGFR inhibition in *EGFR*-mutant NSCLC

**Figure 1:**
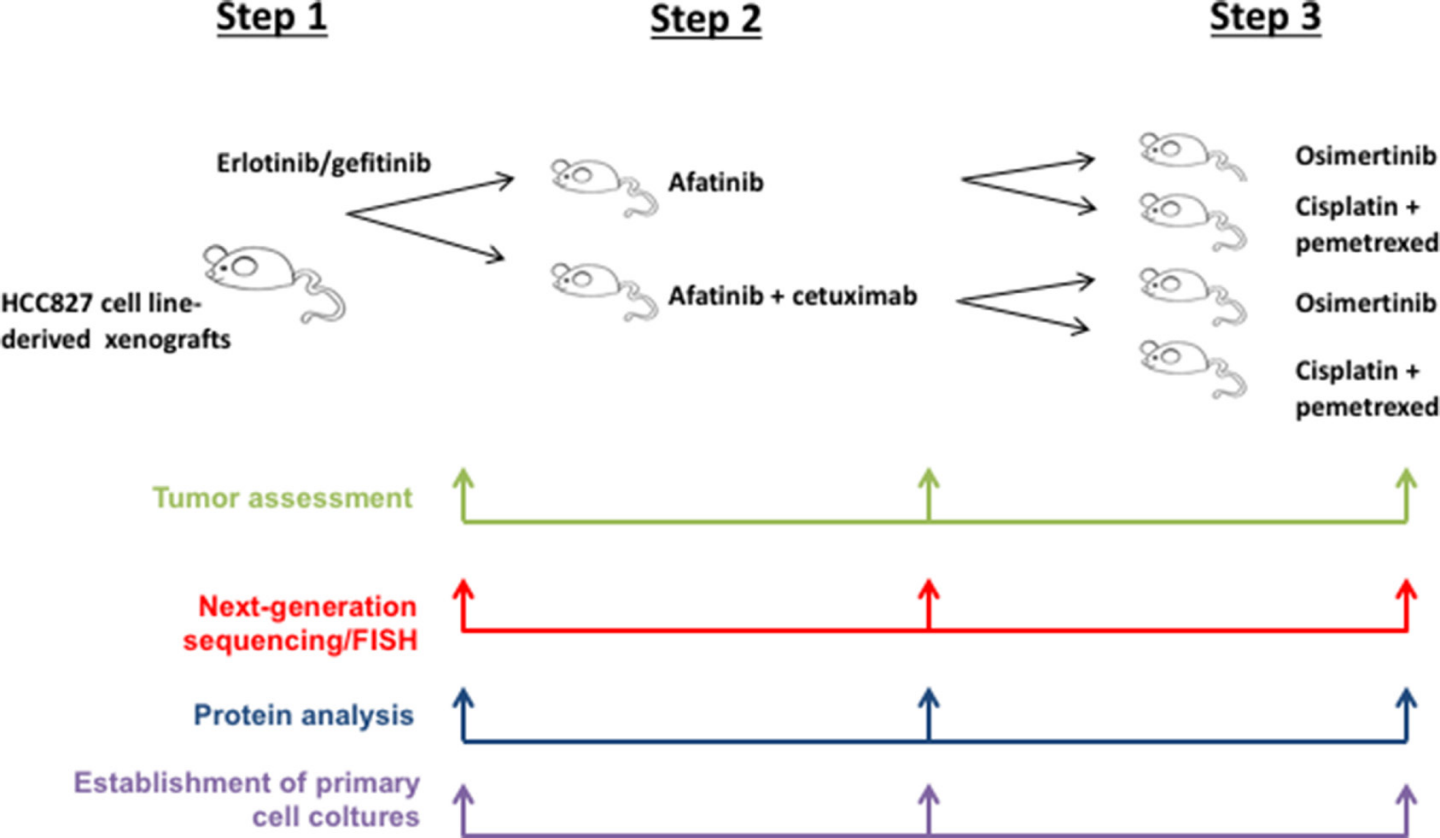
Schematic representation of the whole experiments

An *in vivo* model of EGFR acquired resistance was obtained by treating nude mice xenografted with HCC827, a human NSCLC cell line harboring the *EGFR* activating mutation (del ex19), with a sequence of first-generation EGFR-TKIs (erlotinib and gefitinib) (step 1), second-generation EGFR-TKIs (afatinib) plus/minus cetuximab, anti-EGFR monoclonal antibody (step 2) and third-generation EGFR-TKIs (osimertinib) (step 3) (Figure 1).

**Figure 2:**
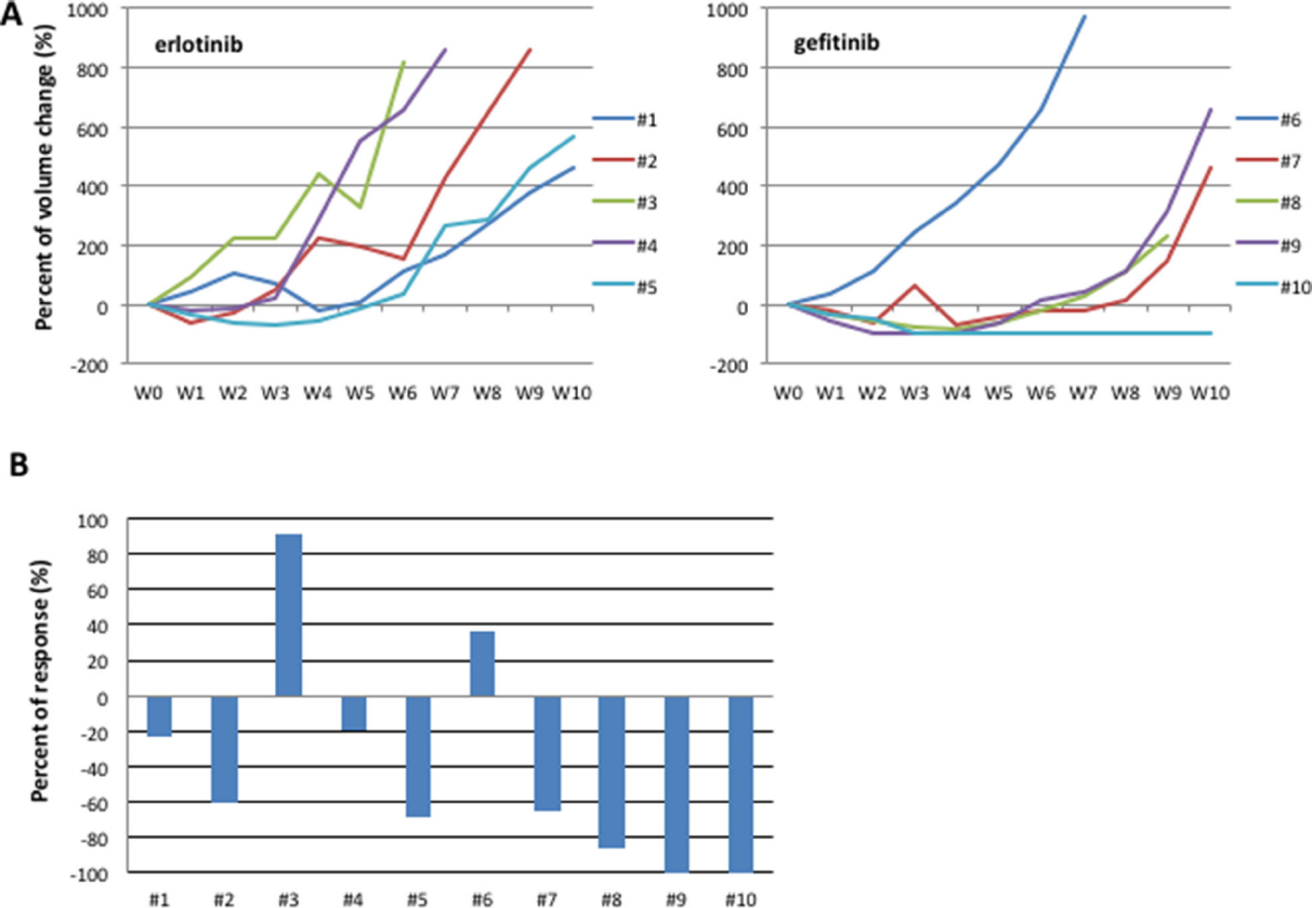
HCC827 human tumor xenografted in nude mice and treated with erlotinib or gefitinib (**A**) Growth curves of tumor volumes in human tumor xenografted in nude mice and treated with erlotinib or gefitinib. CR: complete response (**B**) Best response in HCC827 human tumor xenografted in nude mice and treated with erlotinib or gefitinib.

In the first step, two cohorts of 5 mice each with established HCC827 tumors have been treated with escalating doses of erlotinib or gefitinib over 6 months to derive erlotinib- or gefitinib-resistant tumors (defined as > 25% re-growth from max reduction). For monitoring tumor responses to therapy, we measured volumetric changes and used an arbitrary classification method partially based on clinical research (15): complete response (CR) was defined as no clinical evidence of tumor when mice were sacrificed; partial response (PR) was defined as a decreased of at least 30% in tumor volume with respect to the baseline tumor volume; progression disease (PD) was defined as an increase of at least 20% in the tumor volume with respect to the baseline tumor volume; acquisition of resistance as an increase >25% of re-growth from max reduction; responses that were neither sufficient reduction to categorize regression nor sufficient increase to categorize progression were considered as stable disease (SD). On the basis of this criterion, Figure 2A shows the effect of erlotinib and gefitinib treatment of HCC827 xenograft tumors (10 tumors totally), which resulted in an initial dose-dependent decrease in tumor volume and the subsequent development of acquired resistance in 7/10 tumors and a response rate (RR, PR and CR) of approximately 60%, including one complete response in gefitinib arm, that lasted for 6 months, and a median of duration of response (DoR) of 5 weeks (Figure 2B).

At the end of the treatment period, tumor samples were collected from xenografts by tumor dissection to perform NGS, protein extraction for western blotting analysis, gene amplification analysis by FISH, establishment of *in vitro* primary cell cultures and re-implantation in mice for the following step.

**Figure 3:**
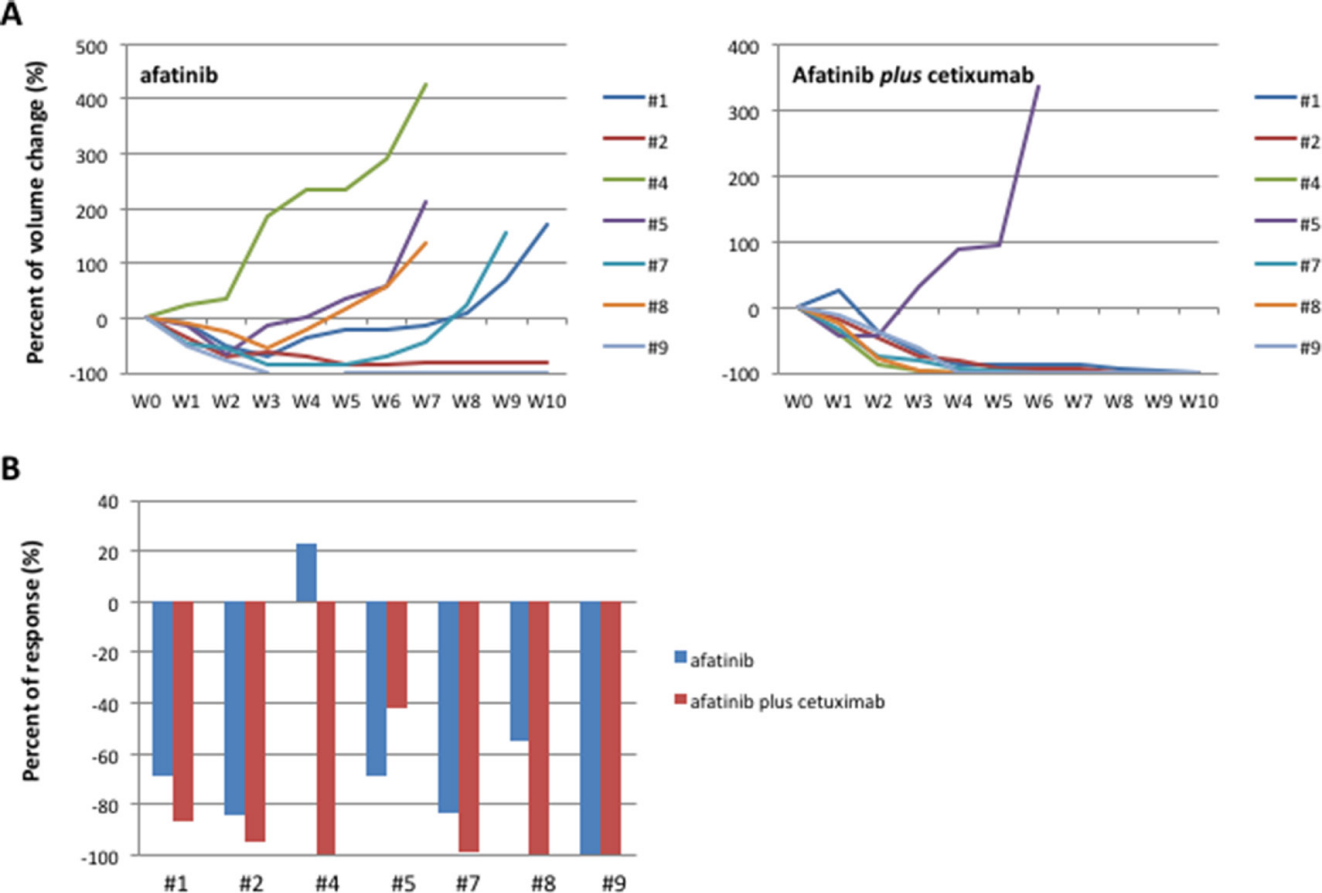
HCC827 human tumor xenografted in nude mice and treated with afatinib or afatinib plus cetuximab (**A**) Growth curves of tumor volumes in human tumor xenografted in nude mice and treated with afatinib or afatinib *plus* cetuximab. (**B**) Best response in HCC827 human tumor xenografted in nude mice and treated with afatinib or afatinib *plus* cetuximab.

To analyze the best therapeutic option for second line treatment, in the second step, each of the 7 first-generation EGFR-TKIs resistant tumors was re-implanted in 2 new mice and randomized to treatment with escalating dose of afatinib or to the combination of cetuximab and afatinib for a total of 14 mice treated in the step 2 (Figure 1, Figure 3A). Before starting treatment with second line drugs, we performed one-week treatment with the respective first-generation EGFR-TKIs used in first line to confirm the persistency of resistance. While afatinib treatment resulted in 5/7 PR, 1/7 CR and 1/7 rapid PD, the combination of afatinib and cetuximab caused a CR in 4/7 tumors and 3/7 PR, with a RR of 100% (Figure 3B). Only one tumor, initially responsive to the combination treatment, displayed a rapid acquisition of resistance. Complete responses to afatinib and to the combination afatinib *plus* cetuximab lasted for more than 6 months. All 5 mice with PR following afatinib treatment experienced progression after a median time of 7 weeks (Figure 3A). Similarly to the first step, tumor samples were collected from the xenografts for molecular analysis, establishment of *in vitro* cultures and for re-implantation.

**Figure 4:**
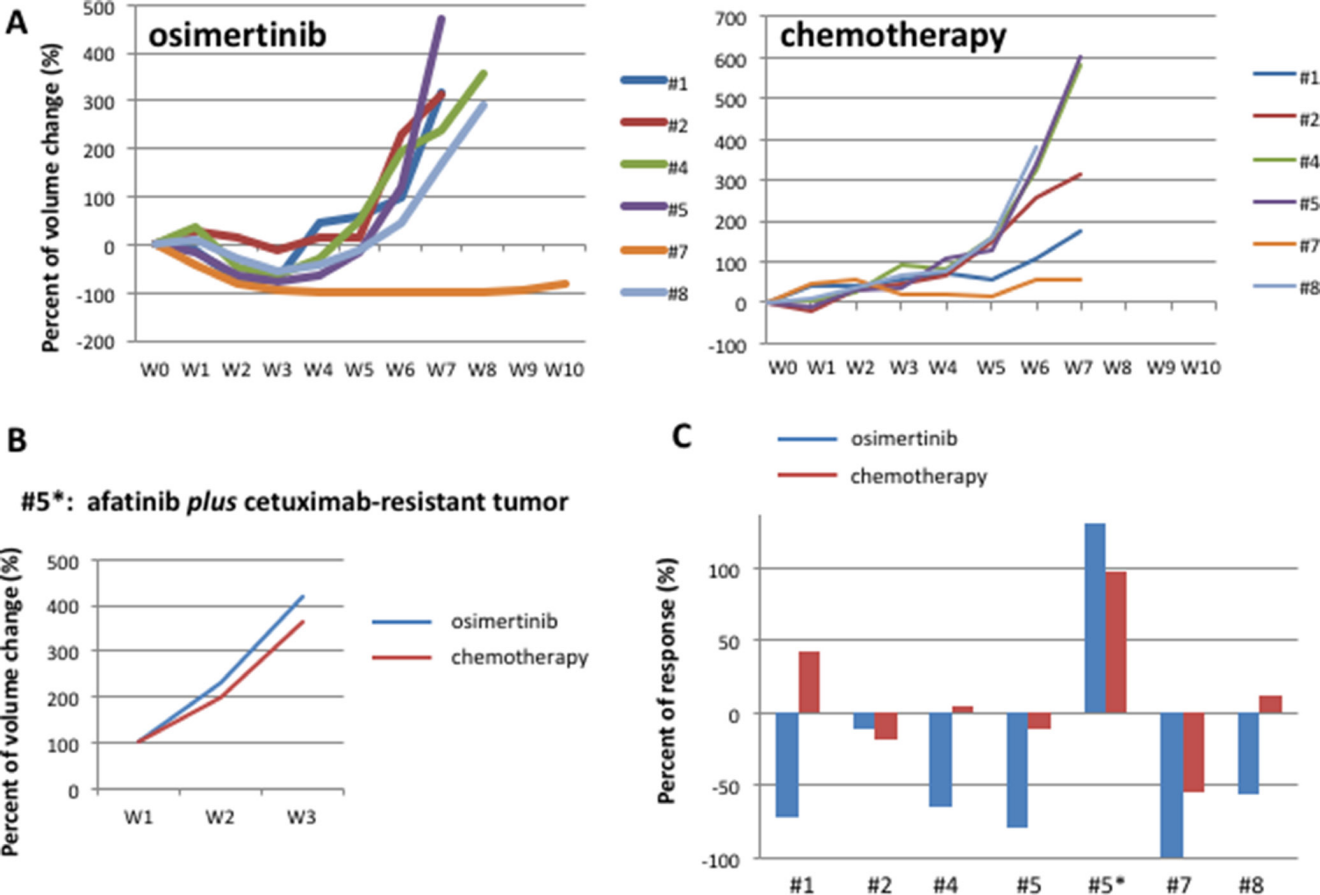
HCC827 human tumor xenografted in nude mice and treated with osimertinib or chemotherapy (**A**) Growth curves tumor volumes in afatinib-resistant human tumor xenografted in nude mice and treated with osimertinib or chemotherapy, represented by cisplatinum *plus* pemetrexed. (**B**) Growth curves of tumor volumes in in afatinib plus cetuximab-resistant human tumor xenografted in nude mice and treated with osimertinib or chemotherapy, represented by cisplatinum *plus* pemetrexed (**C**) Best response in HCC827 human tumor xenografted in nude mice and treated with osimertinib or chemotherapy in third line.

The therapeutic option at progression to afatinib might be represented by standard chemotherapy or by a third-generation EGFR inhibitor [11]. Therefore, in the third step, we decided to re-implant the 5 tumors with acquired resistance to afatinib along with the one tumor with acquired resistance to the combination of afatinib *plus* cetuximab. Each tumor was re-implanted in 2 new mice and randomized to treatment with escalating dose of osimertinib or to standard chemotherapy (Figure 4). Similarly to the previous step, the second line treatment was performed for one week after re-implantion to confirm that tumors were resistant to afatinib or to afatinib *plus* cetuximab. Tumor samples were collected from the xenografts at the end of treatment for molecular analysis and establishment of *in vitro* cells culture. Although none of the 7 EGFR-TKIs resistant tumors presented occurrence of T790M mutation, treatment with osimertinib resulted in a RR of 71 % (including one CR maintained for more than 10 weeks and 4/7 PR) (Figure 4A, 4C). Development of acquired resistance occurred within 7 weeks of treatment. Chemotherapy treatment caused 1/7 PR with rapid development of resistance within 2 weeks and 4/7 SD lasted less than 5 weeks (Figure 4A, 4C). The tumor resistant to the previous treatment with afatinib *plus* cetuximab, and therefore suitable for re-implanation in the third step of experiments, was completely refractory to both osimertinib and chemotherapy treatments (Figure 4B).

NGS analysis on DNA from EGFR-TKIs resistant tumors, collected at each step of resistance acquisition, evidenced a substantial permanence of the driving mutations characterizing the cell line, represented by *EGFR* (E746_A750delELREA) and *KIT* (M541L) mutations, changing from a mean allelic frequency of 99% and 80% respectively in untreated tumors, to 97% and 70% in first-generation EGFR-TKIs-resistant tumors, to 97% and 69% in afatinib- or or afatinib *plus* cetuximab-resistant tumors, and to 98% and 66% in osimertinib-resistant tumors. All samples from mice experiencing acquired resistance did not present the occurrence of T790M mutation. We did not observe the occurrence of new mutations with an allelic frequency higher than 2% with the exception of one single tumor resistant to gefitinib-afatinib-osimertinib, in which the *KRAS*-G12D mutation was detected with an allelic frequency of 8,5%.

### Activation of Hedgehog pathway is a common signature across acquisition of resistance to first-, second- and third-generation EGFR inhibitors

**Figure 5:**
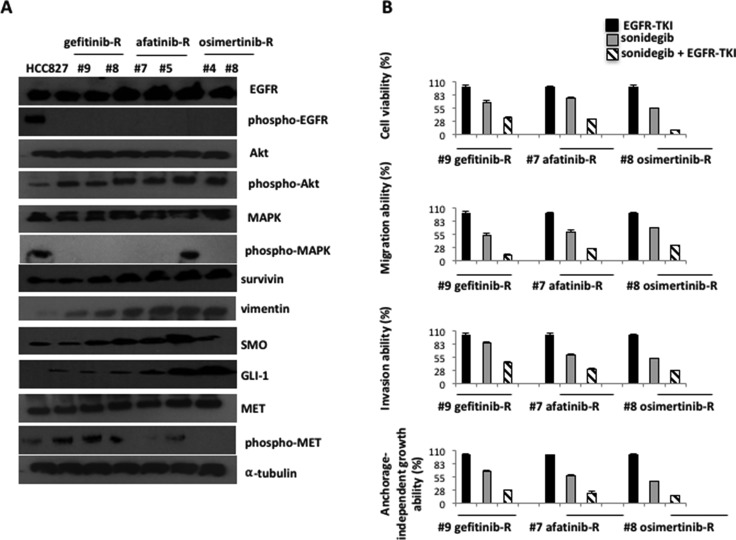
Western blot analysis on protein lysates and experiments on cell lines established *in vitro* from EGFR inhibitors-resistant HCC827 human tumor xenografts (**A**) Western blot analysis on protein lysates from representative tumors of each line of treatment of EGFR-TKIs: gefitinib, afatinib, osimertinib. (**B**) MTT, invasion, migration and anchorage independent growth assays in representative cell lines established *in vitro* from gefitinib-, afatinib-, osimertinib- resistant tumors, treated with the respective EGFR-TKIs, sonidegib and their combination.

Western blot analysis on protein extracts from representative tumors with acquired resistance to first-, second- and third-generation inhibitors showed levels of two key components of Hh pathway, Smoothened (SMO), the 7-membrane-spanning receptor, and GLI1, the principal transcription factor, that progressively increased as compared to untreated controls in the majority of samples (Figure 5A). Activation of MET was evident only in samples from first-generation EGFR-TKIs resistant tumors, whereas all resistant tumors displayed vimentin overexpression indicating the acquisition of mesenchymal properties (Figure 5A). Other receptors, such as AXL and ERBB2, did not show increased activity (Figure 5A). These results confirmed the role of Hh pathway as important mediator of resistance to first generation EGFR-TKIs [6] and revealed that Hh activation is maintained through several lines of therapies with EGFR inhibitors.

In almost all samples derived from different EGFR inhibitor resistant tumors, protein levels of phospho-MAPK were low, with the exception of the one tumor resistant to gefitinib-afatinib-osimertinib, in which we detected the *KRAS*-G12D mutation (Figures 5A, 7A). Conversely, protein levels of phospho-AKT and survivin were increased in all resistant tumors, indicating that, unless new mutation raise during treatment, a common downstream signaling pathway is activated in EGFR inhibitors resistant models (Figure 5A).

To investigate if the activating Hh and MET signals are the consequence of gene amplification, as previously demonstrated [4, 6], we performed FISH analysis on resistant tumor samples, by the use of specific probes for *MET* and *SMO* genes. However, the mean gene copy number of both genes resulted similar in pretreated and resistant samples (data not shown).

Cancer cell cultures established *in vitro* from gefitinib/erlotinib-afatinib-osimertinib-resistant tumor xenografts were used to study the functional significance of increased expression of the Hh pathway components by investigating the effect of SMO inhibition, with the use of a selective SMO antagonist, sonidegib, on cell proliferation and apoptosis, in the presence or absence of EGFR-TKIs (Figure 5B).

Three primary cultures were selected for each step (#9 gefitinib-resistant, #7 afatinib-resistant, #8 osimertinib-resistant). Treatment with sonidegib (1 mmol/L; Figure 5B) alone did not significantly affect the viability of tumor cells. Combined treatment with the respective EGFR-TKI and sonidegib significantly inhibited cell proliferation. Resistant cancer cells have shown an increased expression of vimentin, suggesting that these cells have undergone to EMT. Therefore, we evaluated the abilities of these EGFR-inhibitors resistant cells to invade, migrate, and to form colonies in semi-solid medium *in vitro*. Resistant cells displayed high metastatic abilities that were not affected by the presence of the respective EGFR-TKI (Figure 5B). Thus, we investigated the effect of SMO inhibition, alone or in combination with EGFR-TKI, on cell proliferation and on their metastatic properties. For example, migration ability of EGFR-TKIs resistant cells was significantly affected by the combination of sonidegib with the respective EGFR-TKI, with a reduction to the 12% of gefitinib-resistant cells, and to the 25% of both gefitnib-afatinib- and gefitnib-afatinib osimertinib- cells (Figure 5B). Similary results were obtained for cell proliferation, invasive and anchorage-independent growth abilities (Figure 5B).

**Figure 6:**
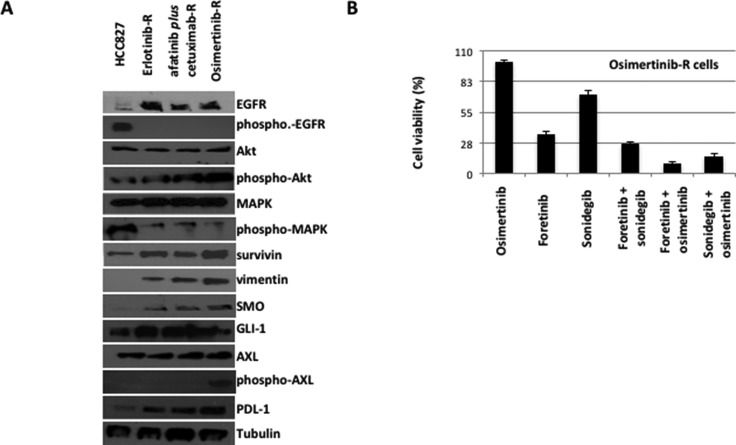
Western blot analysis on protein lysates and proliferation assays on cancer cell lines established *in vitro* from the only one HCC827-xenograft tumor resistant to the sequential treatment with erlotinib, afatinib plus cetuximab and osimertinib (**A**) Western blot analysis (**B**) Cell vailability of erlotinib-afatinib plus cetuximab.osimertinib-resistant cells in the presence of osimertinib, foretinib, sonidegib and their respective combinations.

Of interest, there were two cases of resistant models with activation of peculiar mechanisms. The only single tumor resistant to afatinib plus cetuximab, and then refractory to both osimertinib and chemotherapy, showed AXL activation along with an increased expression of SMO after treatment with osimertinib (Figure 6A). Of interest, PD-L1 protein levels progressively increased with the acquisition of resistance during the three lines of treatment (Figure 6A). Therefore, the cell line established *in vitro* from this osimertinib-resistant tumor xenograft has been used to test the efficacy of the combined inhibition of AXL, by the use of foretinib, and SMO, by the use of sonidegib, on cell proliferation, migration and invasion capabilities of resistant cells, in the presence or absence of osimertinib (Figure 6B). Foretinib resulted a stronger inhibitor of cell proliferation as compared to sonidegib; however the combined treatment of sonidegib or foretinib with osimertinib significantly inhibited cancer cells abilities with a superiority of the combination of foretinib and osimertinib, while the combined blockade of SMO and AXL resulted only in an additive effect (Figure 6B).

**Figure 7:**
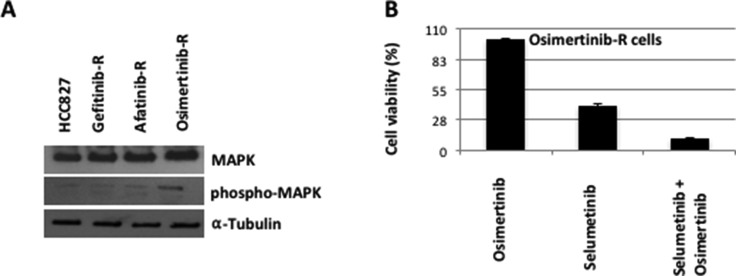
Western blot analysis on protein lysates and proliferation assays on cancer cell lines established *in vitro* from the only one HCC827-xenograft tumor resistant to the sequential treatment with gefitinib, afatinib and osimertinib, harboring the KRAS G12D acquired mutation (**A**) Western blot analysis on protein lysates from gefitinib-, afatinib-, osimertinib-resistant tumors. (**B**) Cell vailability of gefitinib-afatinib-osimertinib-resistant cells in the presence of osimertinib, selumetinib and their combination.

Another particular case was represented by the tumor resistant to gefitinib-afatinib-osimertinib treatment, in which the *KRAS*-G12D mutation occurred. In this tumor protein analysis on the downstream pathway revealed an increased MAPK phosphorylation (Figure 7A). For this reason we decided to test also the activity of the MEK inhibitor, selumetinib, alone or in combination with osimertinib (Figure 7B). Whereas treatment with selumetinib as single agent resulted in a moderate inhibition of cell proliferation, the combination with osimertinib strongly inhibited cancer cell survival (Figure 7B).

## DISCUSSION

NSCLC patients harboring activating mutation of EGFR represent a subgroup of lung cancer patients that can benefit from the treatment with EGFR-TKIs but almost all patients experienced disease progression within 9–12 months from the start of treatment with first generation EGFR-TKIs (erlotinib or gefitinib) [1–3, 16]. More recent data from the randomized phase IIb Lux lung 7 clinical trial [17] confirmed a median PFS of 11 months with the second-generation EGFR-TKI afatinib in first-line treatment. In approximately 50% of cases, acquired resistance to erlotinib or gefitinib involves emergence of the second-site *EGFR* mutation T790M [3]. Other known mechanisms include amplification of the genes encoding the MET and ERBB2 kinases, mutations in *BRAF* or *PIK3CA* [3, 4] and activation of the AXL kinase [5] or of the Hh pathway [6]. Histologic changes such as development of EMT and SCLC features have also been detected in a small subset of tumors from patients with acquired resistance to first-generation EGFR-TKIs [3, 7].

Recently, we demonstrated that gene amplification of *SMO*, which encodes for the receptor of Hh signaling, is a potential mechanism of acquired resistance to first-generation EGFR-TKIs in *EGFR*-mutant HCC827-gefitinib resistant (GR) NSCLC cells [6]. These data are in agreement with the results of a cohort of patients with *EGFR*-mutant NSCLC that were treated with EGFR-TKIs and experienced disease progression [18]. In this cohort, Giannikopoulus and colleagues demonstrated the presence of *SMO* gene amplification in tumor biopsies taken at occurrence of resistance to EGFR-TKIs in 12,5% patients [18] concomitantly with *MET* gene amplification. In this respect, we previously reported that the combined inhibition of both SMO and MET exerted a significant anti-proliferative and pro-apoptotic effect in this model *in vitro* and *in vivo*, with tumor regressions and complete response in 100% of HCC827-GR tumors xenografted in nude mice [6]. Alterations of the *SMO* gene (mutation, amplification, mRNA overexpression) were found in 12.2% of tumors of The Cancer Genome Atlas (TCGA) lung adenocarcinomas by whole-exome sequencing [19]. The incidence of *SMO* mutations was 2.6% and *SMO* gene amplifications were found in 5% of cases. In a small case report series, 3 patients with NSCLC with Hh pathway activation had been treated with the SMO inhibitor sonidegib with a significant reduction in tumor burden, suggesting that Hh pathway alterations occur in NSCLC and could be an actionable and valuable therapeutic target [19].

All these mechanisms can co-exist simultaneously in patients, complicating the overall scenario.

The recent introduction of third-generation EGFR-TKIs in clinical practice encouraged the treatment of EGFR mutant NSCLC patients with multiple lines of EGFR-targeted therapies [20, 21].

Currently, the only available data regarding resistance to the third-generation EGFR-TKI osimertinib [22] were analyzed in AURA clinical study [23, 24] on tissue re-biopsies or circulating tumor DNA samples from resistant patients. These mechanisms include a novel resistance mutation within the *EGFR* gene, the C797S mutation, *HER2* and *MET* amplification and *BRAF* V600E mutation [23, 24].

Considering that therapy with afatinib *plus* cetuximab or osimertinib can be effective in EGFR-TKI resistant tumors [10, 11, 20, 21], the present work studied the efficacy of sequential treatment with various anti-EGFR agents and the molecular mechanisms of tumor evolution and drug resistance in EGFR-mutant lung cancer models. Additionally, the results of this study provided further information on the use of third-generation EGFR-TKIs in T790M negative resistant models and explored new strategies of combined treatment to overcome EGFR resistance. In order to study resistance mechanisms other than the T790M secondary mutation, we chose as preclinical model the HCC827 NSCLC cell line, which is known to activate other signals to escape the EGFR blockade [4–6, 25–27].

Indeed, NGS samples analysis at the time of onset of resistance did not find out the presence of T790M mutation, and confirmed the permanence of the driving mutation of the HCC827 cell line, represented by *EGFR* (E746_A750delELREA) mutation, in all steps, showing the persistency of EGFR activation, and thus the EGFR dependency in these models of resistance, after different lines of treatment. As evidence of this, we demonstrated a significant tumor shrinkage of those tumors resistant to first-generation EGFR-TKIs by using second-generation inhibitors and of those tumors resistant to first- and second-generation EGFR-TKIs with the use of the third-generation inhibitor osimertinib, suggesting that keeping EGFR blockade may represent a valid option of treatment at progression. Furthermore, the combined blockade of the EGFR receptor by an EGFR-TKI and a monoclonal antibody (afatinib *plus* cetuximab) is even more effective. These results further confirm the efficacy of dual vertical targeting of EGFR by the combined treatment of afatinib and cetuximab [9, 10]. A possible reason for this synergism could be found, as previously suggested [9, 10], in the down regulation of the EGFR on the cell surface by cetuximab.

The results of the present study highlighted also the importance of EMT [28, 29] as a crucial event in the acquisition of resistance to second and third-generation EGFR-TKIs inhibitors. Infact, protein lysates from harvested resistant tumors showed higher vimentin levels and EGFR-inhibitors resistant cancer cells established *in vitro* showed high invasive and migrative abilities. Consistently with our previous findings, Hh signaling was confirmed as an important mediator of resistance to EGFR-TKIs inhibitors for first-generation EGFR-TKIs [6, 30–32]. Further, in the present study, we demonstrated that Hh activation is involved also in resistance to second and third generation EGFR-TKIs. The combined blockade of Hh and EGFR by the SMO antagonist, sonidegib, and the respective EGFR-TKI significantly decreased the metastatic behavior of resistant cancer cells established *in vitro* from resistant xenografts, thus revealing that Hh is implicated in the induction of EMT in models of acquired resistance to EGFR inhibitors of first-, second- and third-generation.

Of interest, phospho-MET was confirmed as an important activated signal at resistance to first-generation inhibitor along with SMO activation [6]. However, during the induction of second and third-generation resistant models, MET hyperactivity was lost while SMO activation was maintained through all lines of treatment, suggesting that the Hh pathway was constantly activated over the different treatments and probably was the predominant driver of EMT induced resistance in this model.

One single tumor resistant to the sequential treatment with gefitinib, afatinib and osimertinib showed the presence of *KRAS*-G12D mutation, the only acquired mutation with allelic frequency higher than 2% among all analyzed samples. The contribution of this mutation to the acquisition of resistance was confirmed by the efficacy of combination of the MEK inhibitor selumetinib and osimertinib in the cancer cell line established *in vitro* from this tumor xenograft [22].

The single tumor which was resistant to afatinib plus cetuximab represented a particular EGFR-refractory case. This tumor expressed higher levels of both phospho-AXL and SMO when treated with osimertinib, concomitantly with increased levels of vimentin and PDL-1. Experiments *in vitro* with the cancer cell line, which was derived from this xenograft tumor, showed that the blockade of AXL and SMO, of SMO and EGFR, and, more efficiently, of AXL and EGFR was able to revert the resistance to EGFR. These data highlight the role of AXL and Hh signaling in the mediation of resistance also to second- and third-generation inhibitors. Furthermore, in this model, PDL-1 protein expression increased during the acquisition of resistance. These result support previous data by Lou et al. [33] that investigated the role of EMT on tumor immune microenvironment and found an association between EMT and expression of inflammatory markers, including elevation of PD-L1 expression. They suggested that EMT status and inflammatory microenvironment can be together predictive of EGFR-TKIs resistance and potentially also of responsiveness to new immune checkpoint blockade drugs [33, 34]. Immunotherapic agents, targeting PD-1/PDL-1, recently demonstrated a strong activity in PDL1 positive NSCLC patients in phase II/III clinical trials [35, 36] but they are still little explored in EGFR mutant NSCLC patients. These results warrant further investigation of the role of immunotherapy in EGFR mutant NSCLC that develop resistance to EGFR-TKIs through the acquisition of EMT features [28, 29, 33, 34].

Collectively, the results of the present study confirmed that, even if mechanisms of resistance to first-, second-, third- EGFR-TKIs are heterogeneous, EMT represents a common characteristic of resistant tumors [28, 29]. In this model, EMT is activated together with the Hh pathway in all resistant tumors. The histological change toward mesenchymal phenotype is complex and the mechanisms of its induction are still not fully understood. Hh and AXL pathways are known to play an important role in EGFR resistance [6, 22, 37] through EMT; in one resistant tumor we found that Hh and AXL are concomitantly strongly activated, suggesting that further studies are needed to investigate the interplay between different resistance signaling. Finally, these results encourage a molecular screening of EGFR mutant NSCLC patients at the onset of resistance to design more tailored combination strategy of treatments to prevent and overcome EGFR resistance.

## MATERIALS AND METHODS

### Cell lines and drugs

The human NSCLC HCC827 cell line was provided by American Type Culture Collection (ATCC, Manassas, VA, USA) and maintained in RPMI-1640 (Sigma-Aldrich, Saint Louis, MO, USA) medium supplemented with 10% fetal bovine serum (FBS; Life Technologies, Gaithersburg, MD) in a humidified atmosphere with 5% CO2. The identity of all cell lines was confirmed by STR profiling (Promega, Madison, WV, USA) on an ad hoc basis prior to performing experiments.

The drugs erlotinib, gefitinib, afatinib, LDE-225 (NVP-SONIDEGIB, sonidegib), Foretinib (GSK1363089), Selumetinib (AZD6244), cisplatin and pemetrexed were purchased from Selleck Chemicals (Selleckchem, Houston, TX, USA). Cetuximab was kindly provided by Merck. Osimertinib was generously provided by Astra Zeneca.

### Generation of xenografts in mice of EGFR inhibitors resistant tumors

Four- to 6-week old female balb/c athymic (nuþ/nuþ) mice were purchased from Charles River Laboratories. The research protocol was approved and mice were maintained in accordance with the Institutional Guidelines of the Second University of Naples Animal Care and Use Committee. Mice were acclimatized for 1 week before being injected with cancer cells and injected subcutaneously with 10^7^ HCC827 cells that had been diluted in 200 μL of Matrigel (Corning Life Sciences, MA, USA), 1:1 in culture medium. When tumors reached a mean volume of 150 mm^3^, mice were randomized in two different groups (5 mice/group) of treatments in first step: escalating doses of erlotinib (from 6.25mg/kg/day to 50 mg/kg/day) or gefitinib (from 18.7 mg/kg/day to 150 mg/kg/day) over 6 months to derive erlotinib-/gefitinib-resistant tumors (defined as > 25% re-growth from max reduction). Body weight and tumor volume were monitored on alternate days. Tumor volume was measured using the formula p/6 larger diameter x (smaller diameter)^2^. At the end of treatment period, resistant tumors were re-implated into two nude mice and randomized 1:1 to two groups of treatment (7 mice/group) for second step: first they received again erlotinib or gefitinib to confirm the acquired resistance (and cross-resistance) to first-generation TKIs and then escalating dose of afatinib were administered (6,25 to 25 mg/kg daily, orally) to derive afatinib-resistant tumors or the combination of cetuximab (1 mg/mouse twice per week, intraperitoneally) and afatinib. Similarly, at the end of second line treatment, resistant tumors were re-implanted into two nude mice and randomized 1:1 to two groups of treatment (7 mice/group): an initial treatment with afatinib or afatinib *plus* cetuximab was performed to confirm the acquisition of resistance and then escalating dose of osimertinib (from 5 to 25 mg/kg daily orally) to derive osimertinib-resistant tumors or to standard therapy (schedule: cisplatin from 1 to 3 mg/kg i.p. once a week and pemetrexed from 50 to 150 mg/kg i.p. every four days).

At the end of each step, tumor samples have been also collected from xenografts by tumor dissection to perform next generation sequencing (NGS), protein extraction for western blotting analysis, gene amplification analysis by fluorescence *in situ* hybridisation (FISH), establishment of *in vitro* primary cell cultures, as following described.

### Multiple gene mutation analysis by next generation sequencing (NGS)

DNA extracted from tumor samples harvested form euthanized mice was extracted using the QIAamp DNA Mini Kit (Qiagen) according to the manufacturer's instructions and analyzed with the Ion AmpliSeq Library 96LV Kit 2.0 (Life Technologies) and the Hot Spot Cancer Panel (Life Technologies). This panel gives 154 amplicons covering 2800 mutational hotspot regions in 50 genes (ABL1 EGFR GNAS KRAS PTPN11 AKT1 ERBB2 GNAQ MET RB1 ALK ERBB4 HNF1A MLH1 RET APC EZH2 HRAS MPL SMAD4 ATM FBXW7 IDH1 NOTCH1 SMARCB1 BRAF FGFR1 JAK2 NPM1 SMO CDH1 FGFR2 JAK3 NRAS SRC CDKN2A FGFR3 IDH2 PDGFRA STK11 CSF1R FLT3 KDR PIK3CA TP53 CTNNB1 GNA11 KIT PTEN VHL), with performance of at least 500× sequence coverage for eight samples on one Ion 316 chip [38].

### Protein expression analysis

Tumor samples harvested from mice were cut into 20 mm3 pieces and stored in RNA later until protein extraction for western blot analysis. Protein lysates were obtained by homogenization in RIPA lyses buffer (0.1% sodium dodecylsulfate (SDS), 0,5% deoxycholate, 1%Nonidet, 100mmol/L NaCl, 10 mmol/L Tris–HCl (pH 7.4), 0.5 mmol/L dithiotritol, and 0.5% phenylmethyl sulfonyl fluoride, protease inhibitor cocktail (Hoffmann-La Roche) and clarification by centrifugation at 14,000 rpm for 10 minutes a 4°C. Cancer cells were lysed with Tween-20 lysis buffer (50 mmol/L HEPES, pH 7.4, 150 mmol/L NaCl, 0.1% Tween-20, 10% glycerol, 2.5 mmol/L EGTA, 1 mmol/L EDTA, 1 mmol/L DTT, 1 mmol/L phenylmethylsulfonylfluoride, and 10 μg/mL of leupeptin and aprotinin). Protein lysates containing comparable amounts of proteins, estimated by a modified Bradford assay (Bio-Rad), were subjected to Western blot analysis, as previously described [39]. Immunocomplexes were detected with the enhanced chemiluminescence kit ECL plus, by Thermo Fisher Scientific (Rockford, IL). Desired proteins were probed with corresponding antibodies. Primary antibodies for western blot analysis against p-EGFR (Tyr1068), EGFR, p-MAPK44/42 (Thr202/Tyr204), MAPK44/42, p-AKT (Ser473), AKT, p-MET (Tyr1234/1235), MET, p-AXL (Tyr702), AXL, survivin, SMO, PDL-1, vimentin, GLI1 were obtained from Cell Signaling Technology; monoclonal anti-α-tubulin antibody (T8203) from Sigma Chemical Co. The following secondary antibodies from Bio-Rad were used: goat anti-rabbit IgG and rabbit anti-mouse IgG. Immunoreactive proteins were visualized by enhanced chemiluminescence (ECL plus; Thermo Fisher Scientific). Each experiment was done in triplicate.

### Fluorescence *in situ* Hybridization (FISH) analysis

Tumor samples harvested form euthanized mice were cut into 20 mm3 pieces, formalin-fixed, paraffin-embedded. The slides obtained from paraffin-embedded samples were subsequently hybridized overnight at 37°C with the probes MET (FG0004) and SMO (FA0203) from Abnova, after DNA denaturation at 72°C. Slides were washed with post-hybridization buffer at 72°C, counterstained with 4, 6- diamidino-2-phenylindole (DAPI) and mounted and stored in the dark prior to signal enumeration. For FISH analysis, slides were examined with the Olympus BX41 fluorescence microscope. Areas of optimal tissue digestion and no overlapping nuclei were then selected in each core for counting. Cells [40–60] were counted for each case. We considered cases with green signals of ≥ 4 as amplified.

### Establishment of *in vitro* primary cell cultures

Tumor tissues were minced with scissors in a sterile manner. Tumor cells were dissociated by digestion process with digestion buffer composed by RPMI medium supplemented with Penicillin-Streptomycin and Amphotericin B (Sigma-Aldrich), 1X Collagenase enzyme (Worthington) and 1X Hyaluronidase enzyme (Sigma-Aldrich) in a 37°C shaker at low to moderate speed (e.g. 200 rpm) overnight. Pellets were then resuspended in RPMI-1640 medium supplemented with EGF (Sigma-Aldrich). The cells remained in culture until sufficiently confluent for a first tissue culture passage. A cell culture was considered established if it could be carried through at least 5 *in vitro* passages. However, for establishment of a cell line, cells were maintained in RPMI-1640 medium supplemented with FBS for at least 15 passages.

### Cell proliferation assays

Cancer cells were seeded in 96-multiwell plates and were treated with different doses of indicated drugs for 72 hours. Cell proliferation was measured with the MTT assay, as previously described [39]. IC50 were determined by interpolation from the dose-response curves. Results represent the median of three separate experiments, each performed in quadruplicate. Synergism was calculated with ComboSyn software, ComboSyn Inc., Paramus, NK. 07652 USA.

### Growth in soft agar

Cells (10^4^ cells/well) were suspended in 0.5 mL 0.3% Noble agar (Sigma-Aldrich) dissolved in complete culture medium. This suspension was layered over 0.5 mL 0.8% agar-medium base layer in 12-multiwell plate and daily treated with different concentrations of each drug alone or in combination. When tumor cell colonies were at least 80 μm, they were counted by using a dissection microscope. Assays were performed in triplicate.

### Invasion and migration assays

The *in vitro* invasive ability of cells was measured by using transwell chambers (Corning Life Sciences, MA, USA) according to the manufacturer's protocol. Briefly, cells were seeded onto the membrane of the upper chamber of the trans-well at a concentration of 5 × 10^4^/ml in 500 μl of RPMI medium and were treated with the indicated concentrations of each drug alone and in combination for 24 hours. The medium in the upper chamber was serum-free. The medium at the lower chamber contained 10% FBS as a source of chemo-attractants. Cells that passed through the Matrigel coated membrane were stained with Cell Stain Solution containing crystal violet (Chemicon, Millipore, CA, USA) and photographed after 24 hours. Absorbance was measured at 562 nm by an ELISA reader after dissolving of stained cells in 10% acetic acid. Assays were performed in triplicate.

Cell migration was assessed using a commercially available chemotaxis assay [40]. Assays were performed in triplicate.

### Statistical analysis

The Student *t* test was used to evaluate the statistical significance of the results. All *p* values represent 2-sided tests of statistical significance.
