# Correction: Efficacy of continuous EGFR-inhibition and role of Hedgehog in EGFR acquired resistance in human lung cancer cells with activating mutation of EGFR

**DOI:** 10.18632/oncotarget.28335

**Published:** 2022-12-29

**Authors:** Carminia Maria Della Corte, Umberto Malapelle, Elena Vigliar, Francesco Pepe, Giancarlo Troncone, Vincenza Ciaramella, Teresa Troiani, Erika Martinelli, Valentina Belli, Fortunato Ciardiello, Floriana Morgillo

**Affiliations:** ^1^Oncologia Medica, Dipartimento Medico-Chirurgico di Internistica Clinica e Sperimentale “F. Magrassi e A. Lanzara,” Università degli studi della Campania “Luigi Vanvitelli”, Naples, Italy; ^2^Dipartimento di Sanità Pubblica, Università degli Studi di Napoli Federico II, Naples, Italy

**This article has been corrected:** In Figure 5A, the ‘α-tubulin’ panel is an accidental duplicate of the ‘Akt’ panel. In addition, the ‘GLI-1’ panel has been replaced with a parallel WB evaluating GLI-1 where HCC827 shows the presence of a low level of GLI-1, thus showing 7 lanes instead of 6. The corrected Figure 5A, obtained using the original data, is shown below. The authors declare that these corrections do not change the results or conclusions of this paper.

Original article: Oncotarget. 2017; 8:23020–23032. 23020-23032. https://doi.org/10.18632/oncotarget.15479

**Figure 5:**
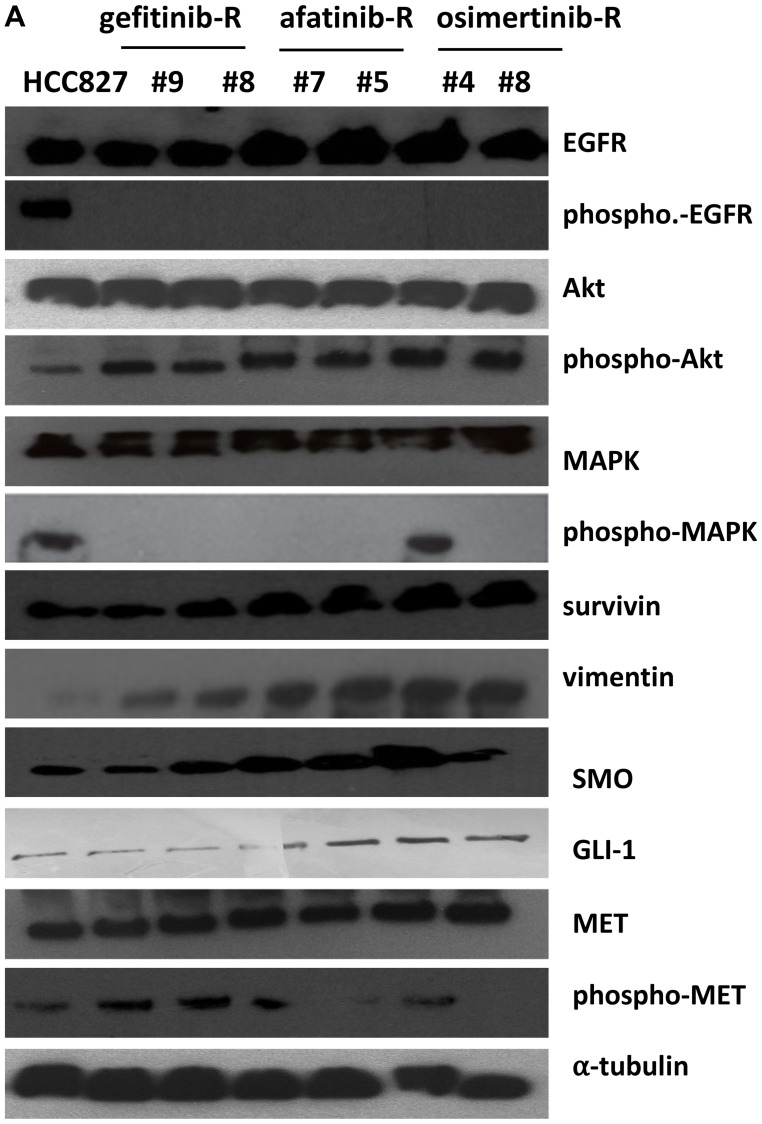
Western blot analysis on protein lysates and experiments on cell lines established *in vitro* from EGFR inhibitors-resistant HCC827 human tumor xenografts. (**A**) Western blot analysis on protein lysates from representative tumors of each line of treatment of EGFR-TKIs: gefitinib, afatinib, osimertinib.

